# Design and Development of Microneedle Electrodes for Automated External Defibrillators

**DOI:** 10.3390/mi17101195

**Published:** 2026-10-16

**Authors:** Dominic Tung, Manoj Sachdev, Sanjit Jolly, Naveen Chandrashekar

**Affiliations:** 1 Mechanical and Mechatronics Engineering, University of Waterloo, Waterloo, ON N2L 3G1, Canada; dominic.tung@uwaterloo.ca; 2 Electrical and Computer Engineering, University of Waterloo, Waterloo, ON N2L 3G1, Canada; msachdev@uwaterloo.ca; 3 Department of Medicine, McMaster University, Hamilton, ON L8P1H6, Canada; sanjit.jolly@phri.ca

**Keywords:** automated external defibrillator, microneedle electrode, transthoracic impedance, electrode–skin resistance, defibrillation

## Abstract

The demand for increased accessibility and effectiveness of automated external defibrillators (AEDs) exists due to the life-threatening nature of sudden cardiac arrest (SCA). Timely access to AEDs can significantly increase the chances of survival in individuals experiencing SCA. Traditional AEDs are bulky and difficult to transport, primarily because of the battery size required to overcome transthoracic impedance (TTI). This study assessed the feasibility of microneedle (MN) electrodes to reduce the TTI and hence improve AED portability. An electrode prototype was developed, consisting of eight arrays of microneedles attached to a 3D printed flexible base. A circuit measuring interelectrode resistance was created to mimic an operational AED while also providing a quantitative measurement of the electrode–skin interface resistance. Testing was conducted on human chest skin. The MN electrode demonstrated a 24% reduction in human transthoracic skin resistance compared to conventional AED pads, suggesting the potential for improved AED effectiveness and portability. These findings indicate the potential for reduced device size, although further research is needed to validate the broader implications.

## 1. Introduction

Sudden cardiac arrest (SCA) requires immediate cardiopulmonary resuscitation and, when a shockable rhythm is present, rapid defibrillation. Out-of-hospital cardiac arrest remains a major public health problem, with survival to hospital discharge remaining low in recent U.S. data [1,2]. Automated external defibrillators (AEDs) make early defibrillation possible outside medical settings.

The limited portability of Automated External Defibrillators (AEDs) remains a challenge, hindering their deployment in non-traditional settings, such as during hiking trips. AEDs weigh between 2.5 and 7 pounds, with volumes ranging from 92 to 570 cubic inches. The primary challenge in weight reduction is the body’s transthoracic impedance (TTI), which disperses a substantial fraction of shock energy across the skin rather than to the heart. TTI affects the current delivered by an external defibrillator. Clinical and review studies show that electrode size, electrode position, and patient factors influence TTI [3,4]. In a recent clinical AED study, smaller 65 cm^2^ electrodes had a median TTI of 100 Ω compared with 88 Ω for 113 cm^2^ electrodes, although first-shock defibrillation success was non-inferior with an impedance-compensating biphasic waveform [3]. The TTI ranges between 35–125 Ω, while delivering 200–360 J of energy using typical electrodes. This necessitates large batteries to ensure that sufficient current reaches the heart muscles, increasing the size and weight of AEDs. If the TTI could be reduced or bypassed, a smaller battery size would be needed to deliver the same amount of shock energy to the heart muscle, resulting in smaller AEDs, improving their portability.

The human skin consists of three layers: the epidermis, dermis, and hypodermis. The epidermis, which is composed of keratinocytes, is highly resistant to electrical currents owing to its tight and dry nature. The outermost layer, the stratum corneum, contains dead keratinocytes that increase electrical resistance [5,6,7]. Bypassing the epidermis could potentially reduce TTI.

While microneedle (MN) technology has been explored for biomedical applications, its efficacy in reducing TTI on human skin for Automated External Defibrillator (AED) systems remains underexplored. It is known that microneedles bypass the high-impedance stratum corneum without skin preparation [8]. Studies comparing traditional wet Ag/AgCl electrodes with MN alternatives for ECG monitoring have concluded that MN electrodes show adequate fidelity for ECG signal detection [9,10,11,12,13,14]. For long-term monitoring, MN electrodes demonstrate higher-amplitude performance and accuracy than conventional electrodes [15,16]. Kitamura et al. and Tezuka et al. showed that their MN device reduced the threshold voltage for tactile receptor stimulation by 70–85% versus conventional electrodes [17,18]. A transcutaneous electrical nerve stimulation study showed reduced electrode–skin impedance [19].

Conductive microneedle electrodes are therefore an established strategy to reduce the electrical barrier of the stratum corneum in both recording and stimulation applications. Griss et al. fabricated silicon microneedle arrays coated with Ag/AgCl and demonstrated reduced electrode–skin impedance for biopotential recording [20]. Flexible implementations have since been reported using Ti/Au-coated microneedles on PET substrates [21], conductive fabric-backed polymer microneedles [22], and Ti/Au/PEDOT-coated polyimide arrays [23]; these devices achieved low electrode–skin impedance and supported ECG, EMG, EEG, or related electrophysiological recording. A recent microneedle/hydrogel TENS system similarly used microneedles to penetrate the stratum corneum and establish a low-resistance pathway for electrical stimulation [24]. Collectively, these studies show that the impedance-reducing function of conductive microneedles is well established outside defibrillation. However, AED specific application of microneedles is not well researched.

Previous studies have shown that electrode size and placement affect the TTI. Dalzell et al. reported lower TTI with larger defibrillation pads and higher success with low-energy monophasic shocks [25], while Garcia and Kerber found that clinically recommended pad positions produced similar TTI values [26]. More recently, Yin et al. reported higher TTI with smaller AED electrodes, but similar defibrillation success when impedance-compensating biphasic waveforms were used [3]. These studies used conventional adhesive electrodes and did not evaluate microneedle interfaces. The gap addressed in the present study is therefore limited but specific: whether a microneedle interface can be incorporated into an AED-type pad and reduce measured resistance in human cadaveric chest tissue. We hypothesized that the MN electrode would show lower electrode–skin resistance than a conventional AED electrode under matched test conditions.

## 2. Materials and Methods

**Figure 1:**
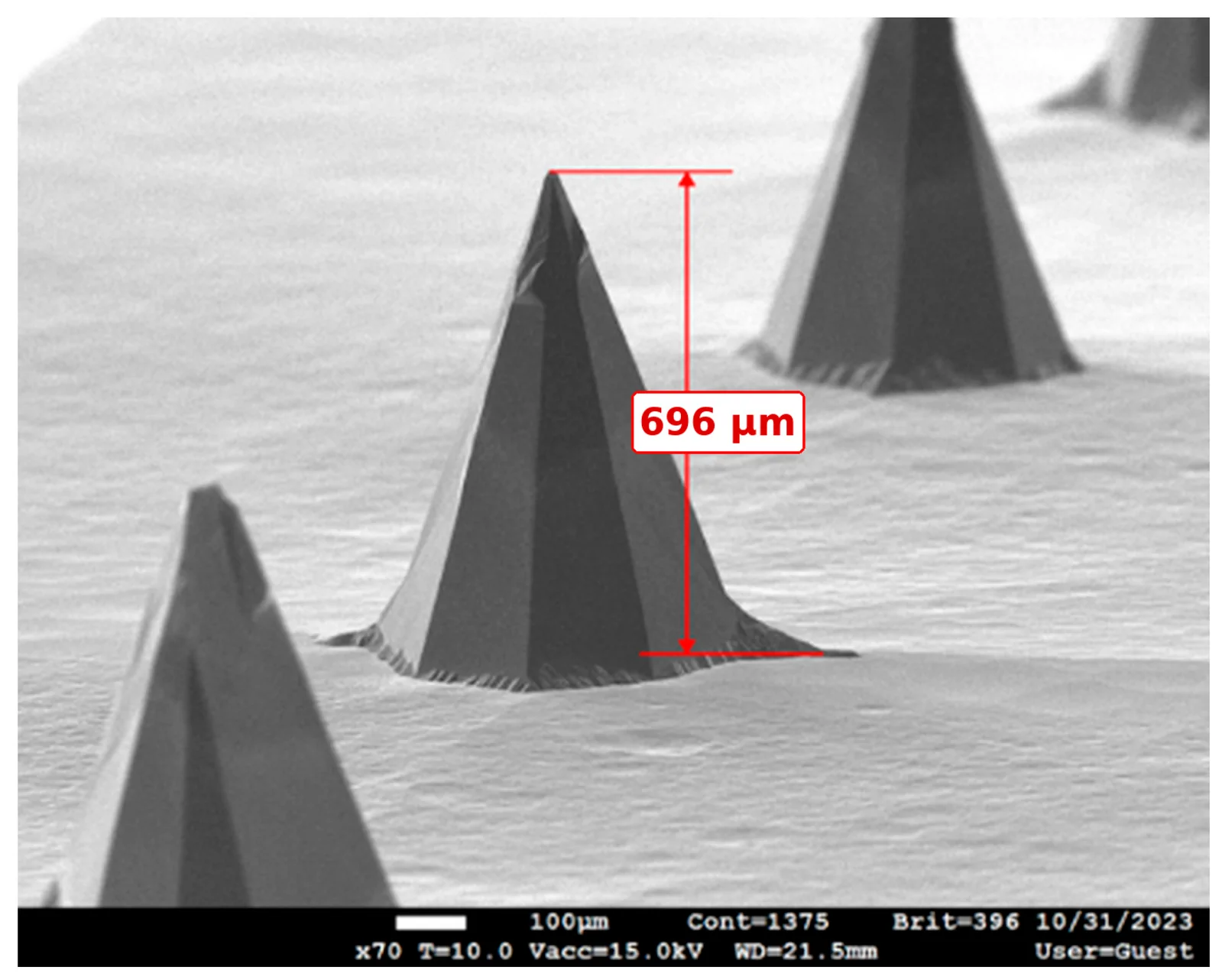
SEM image of microneedle array. The measured needle height is approximately 696 µm (0.7 mm).

The microneedle arrays were fabricated from a 1 mm thick, 4-inch (100) silicon wafer using bulk micromachining [27,28]. The wafer was first cleaned with acetone under ultrasonication for 5 min. A 200 nm silicon nitride (SiN) layer was then deposited on both sides of the wafer by low-pressure chemical vapor deposition (LPCVD) and used as the main masking layer. The microneedle pattern was defined using AZ nLOF 2035 photoresist and UV lithography, followed by reactive ion etching to transfer the pattern into the SiN layer. Before wet etching, the wafer was mechanically diced into individual cluster regions. The microneedles were formed by anisotropic wet etching in 29 wt% potassium hydroxide (KOH) at 80 °C for approximately 10 h. After etching, the arrays were cleaned using an RCA-1 process for 5 min at 75 °C. A 50 nm titanium (Ti) adhesion layer was then deposited, followed by a 50 nm gold (Au) layer, using electron-beam evaporation at a university microfabrication facility. The 50 nm Ti thickness was part of the fabrication process used by the facility and was not optimized as a study variable. During metal deposition, the wafer was kept in the standard horizontal orientation; it was not deliberately tilted or rotated, and deposition was not repeated from multiple angles. SEM measurements of the completed arrays indicated an average needle height of approximately 700 µm, a tip diameter of approximately 3 µm, and a needle pitch of 2.2 mm (Figure 1).

**Figure 2:**
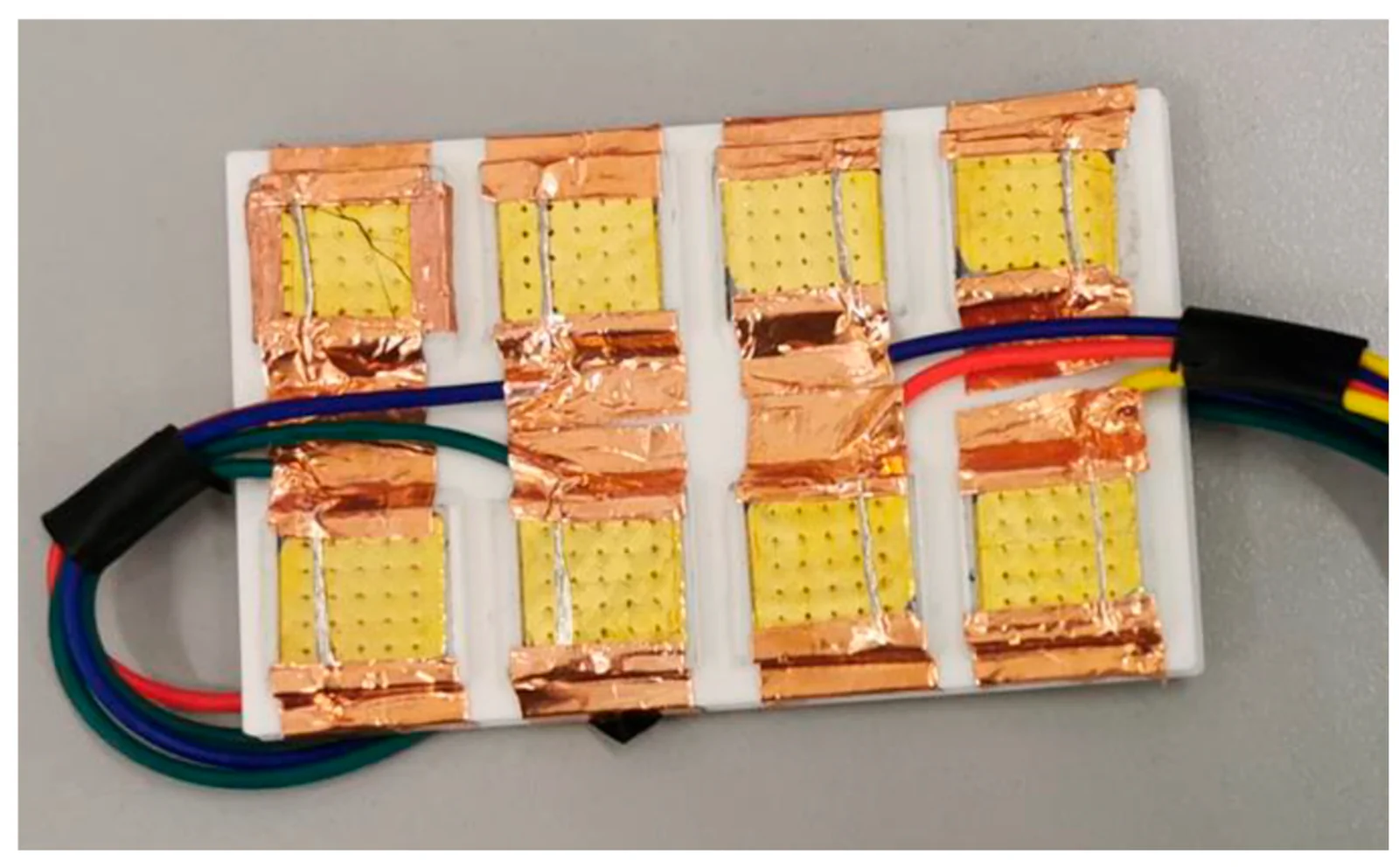
Microneedle pad prototype.

Each microneedle cluster had 25 microneedles, measured approximately 1.3 × 1.3 cm, with the active microneedle array occupying a smaller region of the cluster. The eight clusters fabricated were attached to a 3D-printed flexible thermoplastic polyurethane (TPU) base for surface conformity. The microneedles themselves were silicon; TPU was used only as the flexible support for the rigid silicon clusters. TPU has also been used in biomedical and implantable device fabrication [29,30]. The purpose of the TPU in this prototype was therefore mechanical support and surface conformity, rather than formation of the microneedle tips. Owing to EBPVD limitations, surface wiring was used for connections, with a 24-gauge wire connected to each cluster using copper adhesive (Figure 2). The wiring connected to the terminal block served as the electrode lead, with a system resistance between 0.9 and 2.3 Ω.

**Figure 3:**
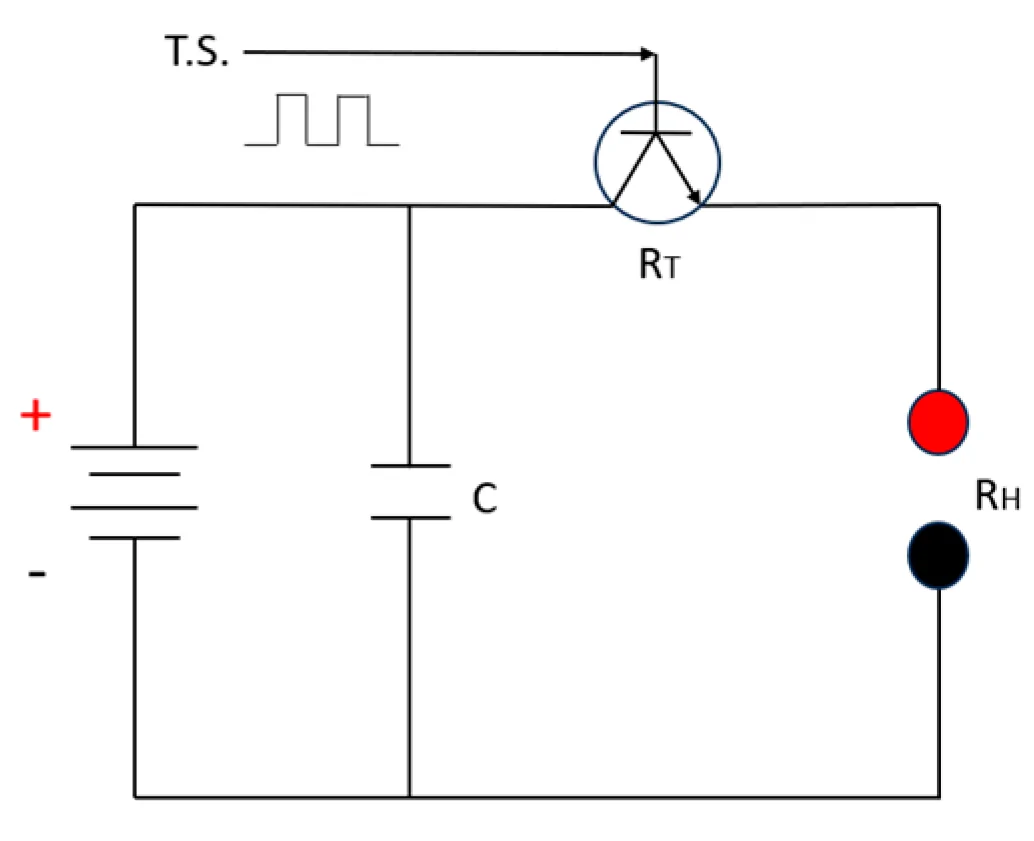
Defibrillator power circuit [31].

To compare interelectrode resistance with the MN and conventional electrode configurations, a low-voltage RC measurement circuit (Figure 3)was developed based on the analytical approach described by Kugelberg [31]. The circuit used a DC-powered parallel RC circuit to simulate AED behavior during defibrillation and measure electrical resistance over the human myocardium [31]. The procedure delivered a square electrical pulse through a capacitor, which was controlled via a transistor to manage the pulse duration and interval. It was not intended to reproduce a clinical biphasic defibrillation waveform and did not deliver therapeutic defibrillation energy. The circuit model is illustrated in Figure 3. Here, T.S. is the signal trigger, C is the capacitor, RT is the transistor switch, and RH is the resistance in the measured electrical path.

The resistance can be calculated by measuring the voltage differential across the resistance and the discharge time. Equation (1) describes the circuit behavior, where V2 and V1 are the voltages (V), T is the pulse duration (s), C is the capacitance (F), and R is the resistance (Ω) [31].

(1) $$\frac{V_{2}}{V_{1}}=e^{-\frac{T}{\mathrm{RC}}}$$

**Figure 4:**
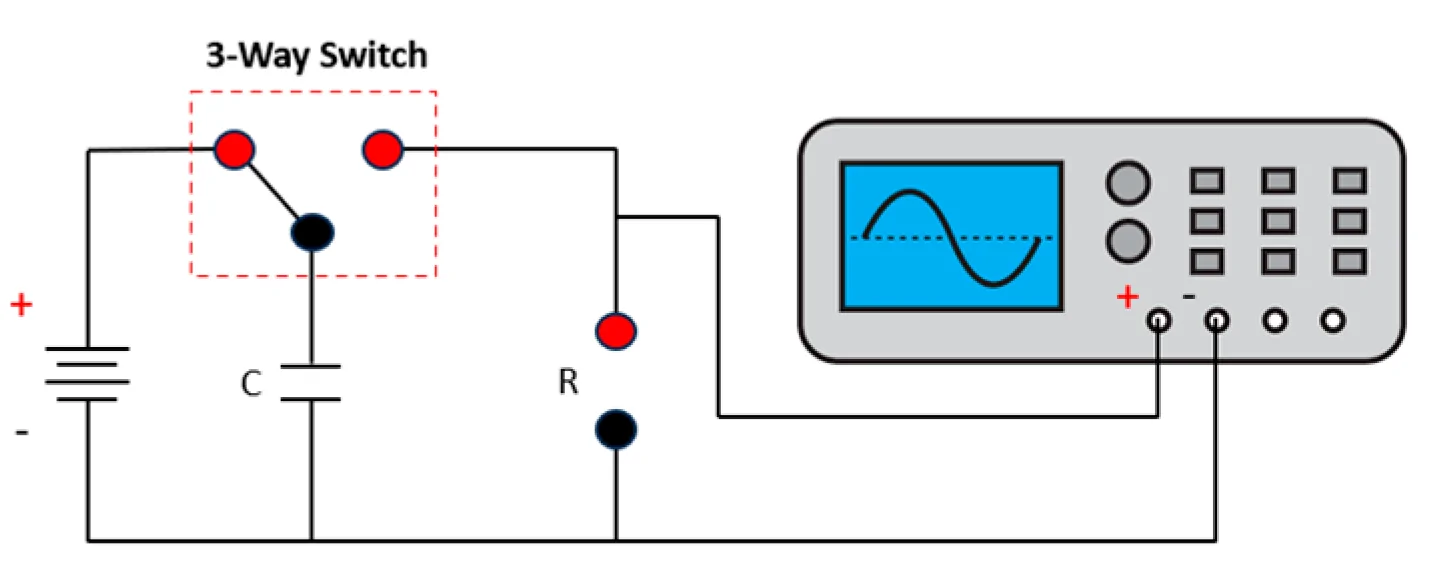
Experimental test circuit model.

Based on this model, an experimental circuit was developed with a DC power supply (5 V), three-prong switch as the control transistor, capacitor (100 μF), wires, and breadboard (Figure 4). A manual state trigger was used to capture the single trigger instances. The experimental test circuit model is shown in Figure 4. By isolating Equation (1) in terms of resistance, the interelectrode resistance can be calculated as shown in Equation (2).

(2) R(ohms)=T(ms)×10−3[C(μF)×10−6]×[ln V1(volts)V2(volts)]

The functionality of the MN electrode pad was evaluated using a 90-year-old male unembalmed human cadaver. The body had never been frozen and was stored in a refrigerated morgue at 2–4 °C until the experiment. The surface of the body was acclimated to room temperature for 24 h prior to testing. This test used one donor and was intended as a feasibility test rather than a population-level comparison.

**Figure 5:**
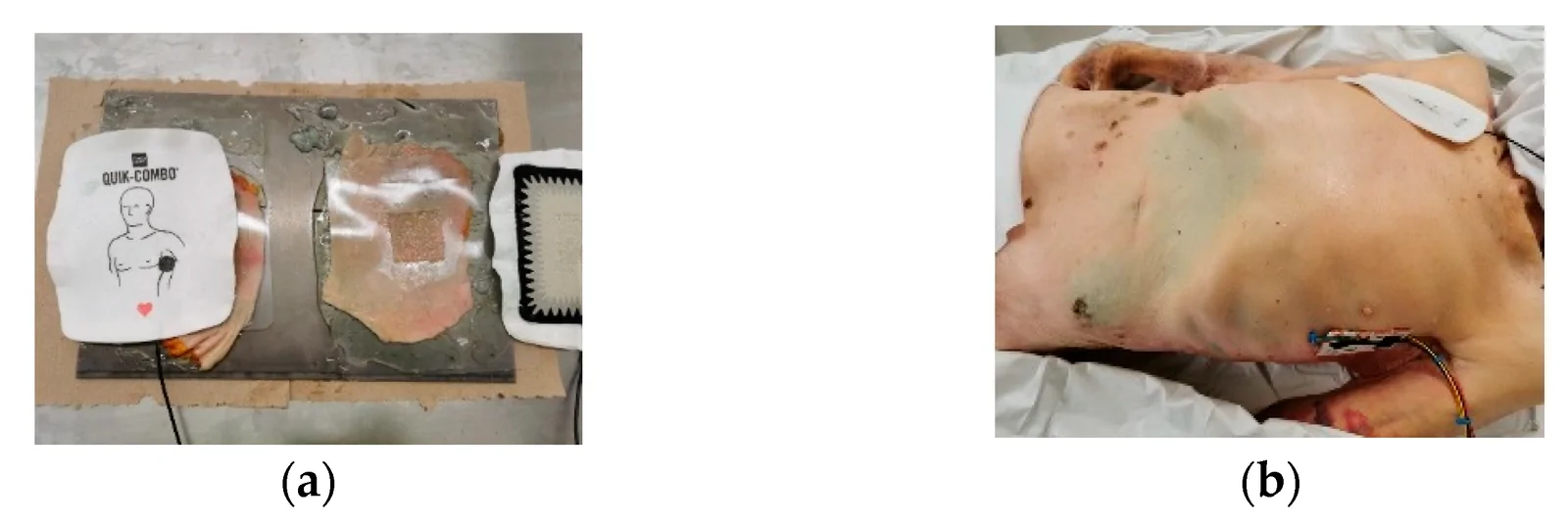
(**a**) Testing using harvested skin. (**b**) Testing using intact cadaver.

The pads were placed diagonally on the front side of the chest, one below the clavicle on the sternum and the other placed laterally, as shown in Figure 5. First, both terminals were connected to conventional pads, and the resistance was measured. Then, the circuit’s positive terminal was connected to the MN pad prototype with eight clusters, with the conventional pad as the ground terminal, and the resistance was calculated using Equation (2). The eight-cluster MN prototype represented about 8/67 (approximately 12%) of the active area of the full conventional pad based on the cluster-equivalent area used in the original masking method. Therefore, this intact-cadaver test was not area matched and was used only to confirm that the MN prototype could reduce the resistance.

The MN electrode was also evaluated on chest skin harvested from one recently deceased 96-year-old female donor. The body was stored at 2–4 °C and allowed to reach room temperature for 24 h before testing. Skin was harvested from two chest sites corresponding to normal AED pad locations and placed on an aluminum plate to provide a common conductive backing (Figure 5). The same excised-skin/aluminum setup was used for the conventional and MN measurements. The conventional pad was masked so that its exposed conductive area matched the footprint represented by 2, 4, 6, or 8 MN clusters. The full conventional pad corresponded to approximately 67 cluster-equivalent footprints. The original experiment recorded area in cluster-equivalent units; the absolute active area in cm^2^ was not recorded and cannot be reported reliably after the fact. Interelectrode resistance for each condition was calculated using Equation (2).

## 3. Results

**Figure 6:**
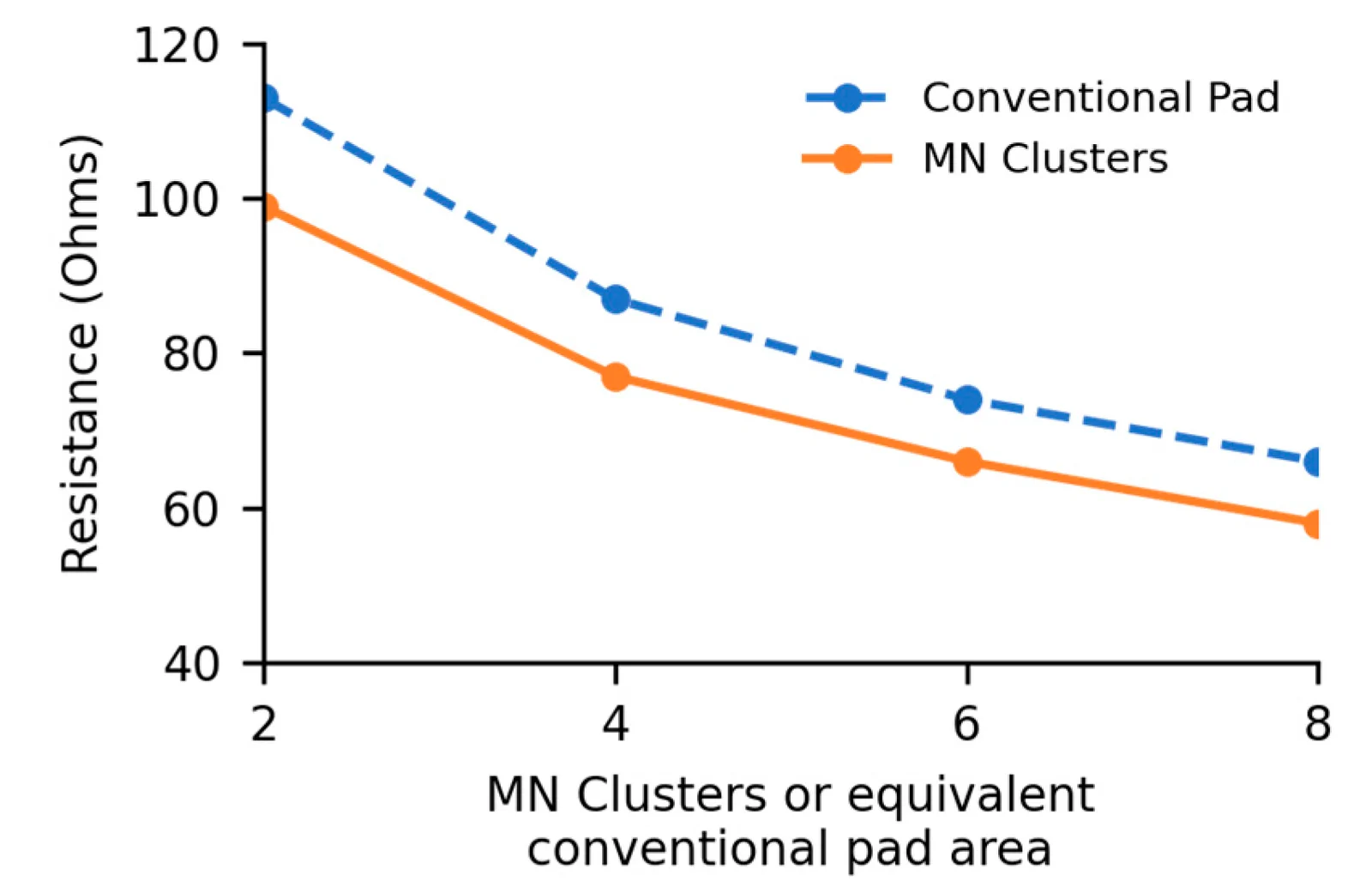
Effect of exposed electrode area on interelectrode resistance in the excised-skin/aluminum-plate test. The dotted line represents the area-matched conventional pad condition and the solid line represents the MN condition.

In the intact-cadaver test, two full-size conventional pads produced a measured resistance of 858 Ω. Replacing one full-size conventional pad with the eight-cluster MN prototype produced 878 Ω. Because the MN prototype had only about 8/67 of the active area of the full conventional pad, these two values are not an area-matched performance comparison. In the separate excised-skin/aluminum-plate test, the full conventional the aluminum plate had a resistance of 37 Ω. The 37 Ω value hence represents the resistance of the test media. For the area-matched 2-, 4-, 6-, and 8-cluster comparisons, resistance decreased as exposed area increased for both electrode types (Figure 6). At matched exposed area, the MN condition was approximately 12% lower than the conventional condition in this donor. If a similar reduction occurred at both electrode–skin interfaces, a two-MN-pad configuration would correspond to a projected reduction of approximately 24%.

## 4. Discussion

This study was intended as an early proof-of-concept test of an AED-specific microneedle interface. The primary goal of this research was to investigate whether a microneedle AED pad would be effective in decreasing the resistance offered by the epidermal layer of the skin and the magnitude of such decrease. The microneedles were fabricated on an inflexible substrate. Therefore, a large MN pad from a single substrate would result in an inflexible pad. To address this issue, several small clusters of microneedles were fabricated and bonded onto a flexible plastic substrate. Owing to resource constraints, only one MN electrode, which was one-eighth the size of a conventional electrode, was manufactured. The purpose of testing the cluster on an intact cadaver was to demonstrate the suitability of using the MN pad in a natural environment. The intact-cadaver test showed that the prototype could be used in the full-body measurement setup, but it was not area matched: the MN prototype represented only about 8/67 of the active area of the conventional pad. The 858 Ω versus 878 Ω intact-cadaver values therefore should not be interpreted as evidence that the small MN prototype performs the same as a full conventional pad. To test the reduction in resistance due to MN pad and to remove variables such as unknown core body temperature in an intact cadaver, tests were run on only the chest skin using conventional pads of various sizes and different numbers of MN clusters. Both pads showed similar trendline characteristics, with consistent rates of decrease in resistance when the pad area was increased. As there was no indication of diminished relative returns between the pads, the separation pattern remained consistent (Figure 6). The results indicate that an equivalent sized MN electrode pair would show a 24% improvement in reducing human TI compared to the conventional AED electrode.

The contribution of the present work is not the general use of conductive, metal-coated, or flexible microneedles, which has already been reported for recording and stimulation applications. The specific contribution is the adaptation of metallized silicon MN clusters to an AED-type electrode interface and their preliminary evaluation on human cadaveric chest tissue. The study uses a low-voltage RC resistance-measurement circuit to test whether bypassing the stratum corneum changes the resistance measured between the two electrode locations. It does not test a clinical defibrillation waveform or defibrillation success. Although the magnitude of impedance reduction reported in the present study is smaller, the use of shorter and finer microneedles represents a more clinically realistic and patient-compatible approach for future AED electrode designs. Further research is needed to find what weight reduction in battery is possible if 24% reduction in TTI is possible using full sized MN pads.

The present results should also be viewed in the context of conventional AED electrode studies. Dalzell et al. showed that larger electrode pads reduced TTI and improved success with low-energy monophasic shocks [25]. In a more recent clinical AED study using impedance-compensating biphasic waveforms, Yin et al. found that smaller electrodes increased TTI from a median of 88 Ω to 100 Ω, but first-shock defibrillation success remained non-inferior [3]. Garcia and Kerber also showed that recommended pad positions can produce similar TTI values [26]. These studies show that electrode resistance is only one part of defibrillation performance. The lower resistance observed with the MN interface in the present study cannot be taken as evidence of improved defibrillation success. However, the lower resistance could mean a smaller battery would suffice if other variables are constant.

The main study limitations are the use of one intact cadaver and one excised-skin donor, lack of repeated specimens and inferential statistics, post-mortem tissue, the low-voltage RC test circuit rather than an actual biphasic defibrillation waveform, lack of controlled contact pressure, and the absence of two full-size MN pads. The results should therefore be interpreted as preliminary feasibility data. Safety and usability were not evaluated in this cadaveric study. The silicon microneedles were approximately 0.7 mm high with a very small tip diameter, but the study did not measure pain, bleeding, skin trauma, infection risk, microneedle breakage, or difficulty with placement and removal. It also did not evaluate use by laypersons in an emergency. These issues are important for an AED electrode and must be studied before any clinical use is considered. Future work should include mechanical integrity testing, inspection for broken needles, tissue-damage assessment, and usability testing in addition to electrical testing. A further fabrication limitation is that the Ti/Au coating was not optimized.

All values are individual measurements from this proof-of-concept study; no population mean, standard deviation, confidence interval, or inferential statistical test is reported. The 858–878 Ω intact-cadaver values and the lower excised-skin values represent different electrical paths and should not be compared directly. Clinical defibrillator studies commonly report TTI values on the order of roughly 70–110 Ω depending on pad size and position [3,25,26], but those clinical measurements are also not directly comparable with the present low-voltage post-mortem RC measurements. A larger paired study using full-size electrodes, controlled pressure and placement, repeated measurements, multiple donors, and clinically relevant defibrillation waveforms is needed before quantitative performance claims can be made.

## 5. Conclusions

This preliminary study evaluated the feasibility of using a microneedle (MN) interface in an automated external defibrillator (AED) electrode. In the area-matched excised-skin test, the MN condition showed lower interelectrode resistance than the conventional electrode condition. Extrapolation of the observed single-interface difference to two MN interfaces gave a projected resistance reduction of approximately 24%, but this value was not directly measured using two full-size MN pads. The results therefore support further investigation of the MN electrode concept. Future work should use full-size MN electrodes, multiple donors, controlled contact conditions, repeated measurements, biphasic defibrillation waveforms, and dedicated safety and usability testing.

## Data Availability

All data from the study are available from the corresponding author on reasonable request.
